# Supplementary material for: Nucleotide degradation and ribose salvage in yeast
Source: Mol Syst Biol. 2013 May 14;9:665. doi: 10.1038/msb.2013.21 (PMC4039369; doi:10.1038/msb.2013.21)
Supplement: Supplementary Information — Supplementary Figures S1–4, Supplementary Tables S1–3 [file msb201321-s1.pdf]

Nucleotide degradation and ribose salvage in yeast

Yi-Fan Xu[1,2], Fabien Létisse[4], Farnaz Absalan[3], Wenyun Lu[1], Ekaterina Kuznetsova[5], Greg Brown[5], Amy A. Caudy[6], Alexander F. Yakunin[5], James R. Broach[3], Joshua D. Rabinowitz[1,2*]

[1]Lewis Sigler Institute for Integrative Genomics, Princeton University, Princeton, NJ 08544, USA.

[2]Department of Chemistry, Princeton University, Princeton, NJ 08544, USA.

[3]Department of Molecular Biology, Princeton University, Princeton, NJ 08544, USA

[4]Université de Toulouse, INSA, UPS, INP; LISBP, 135 Avenue de Rangueil, F-31077 Toulouse, France

[5]Department of Chemical Engineering and Applied Chemistry, Banting and Best Department of Medical Research, University of Toronto, Toronto, ON M5S 3E5, Canada

[6]Donnelly Centre for Cellular and Biomolecular Research, University of Toronto, Toronto M5S 3E1, Canada

*To whom correspondence should be addressed. E-mail: joshr@princeton.edu

Supplemental Figures

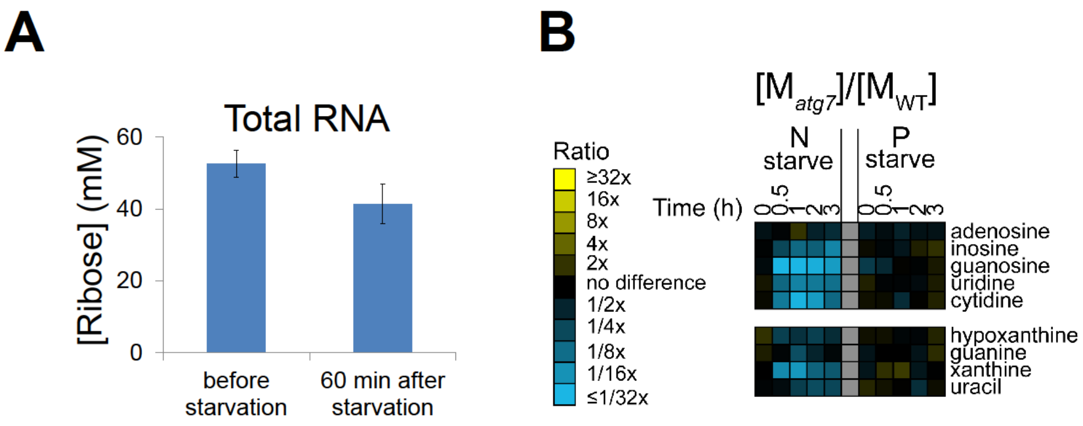

Figure S1. Macroautophagy triggers RNA degradation

(**A**). Total RNA level before and 1 hour after carbon starvation. The *y* axis represents RNA level in the units of ribose concentration (mean ± range of N = 2 biological replicates). (**B**). Ratio of metabolite level in *atg7* strain vs. wild type strain in nitrogen and phosphate starvation. All reported values are log_2 transformed ratios; data are mean of duplicate samples at each time point.

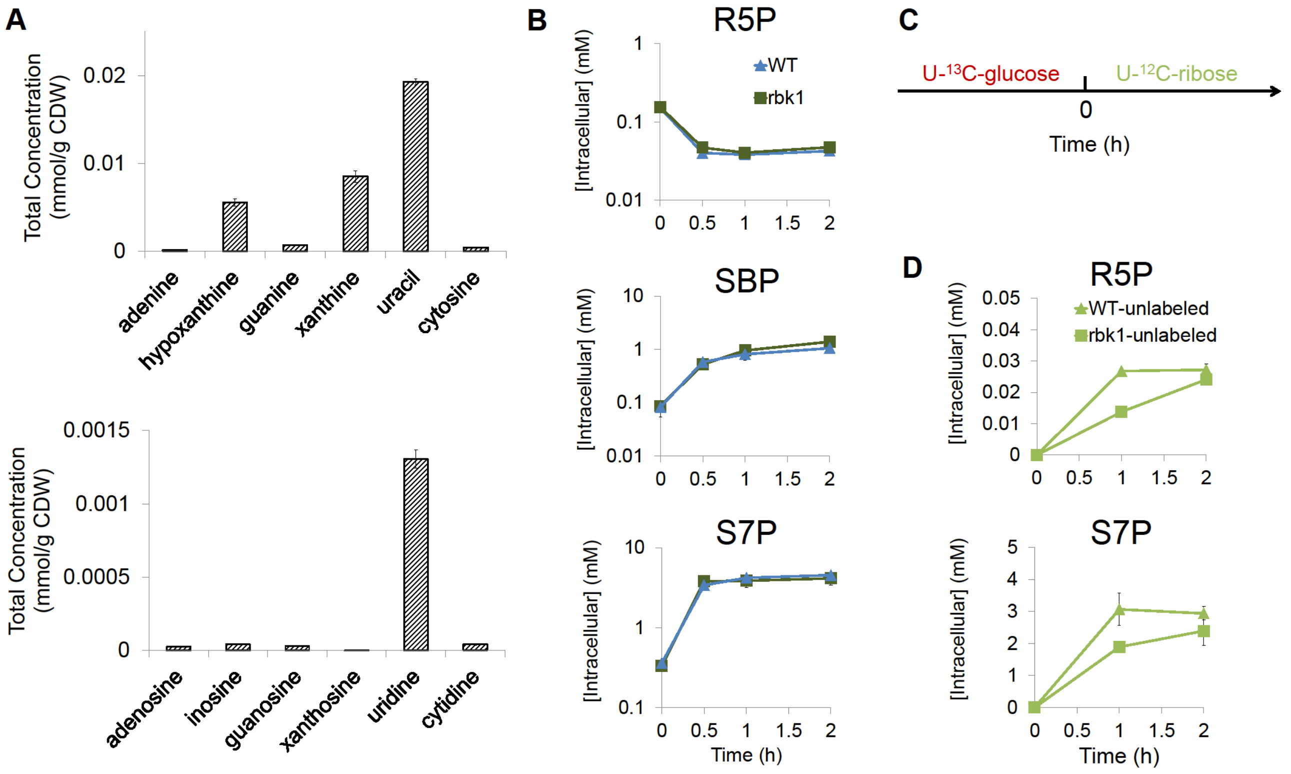

Figure S2. Clarification of downstream steps in ribose salvage pathway

(**A**). In carbon starvation, the total concentration of guanine, cytosine and cytidine is much lower than the concentration of their deaminated form. The *y* axis represents the sum of intracellular and extracellular metabolite amount divided by cell dry weight (mean ± range of N = 2 biological replicates) at 1 hour after carbon starvation. (**B**). *rbk1* deletion did not change the concentration of pentose phosphate intermediates following carbon starvation. (**C**). Experimental design of data shown in (**D**). Yeast cells growing on 2% U-^{13}C-glucose were switched to 2% U-^{12}C-ribose, and metabolome was analyzed by LC-MS. (**D**). The increase of unlabeled R5P and S7P made from U-^{12}C-ribose. In (**B**) and (**D**), the *x* axis represents hours after glucose removal, and the logarithmic (**B**) and linear (**D**) *y* axis represents absolute intracellular concentration (mean ± range of N = 2 biological replicates).

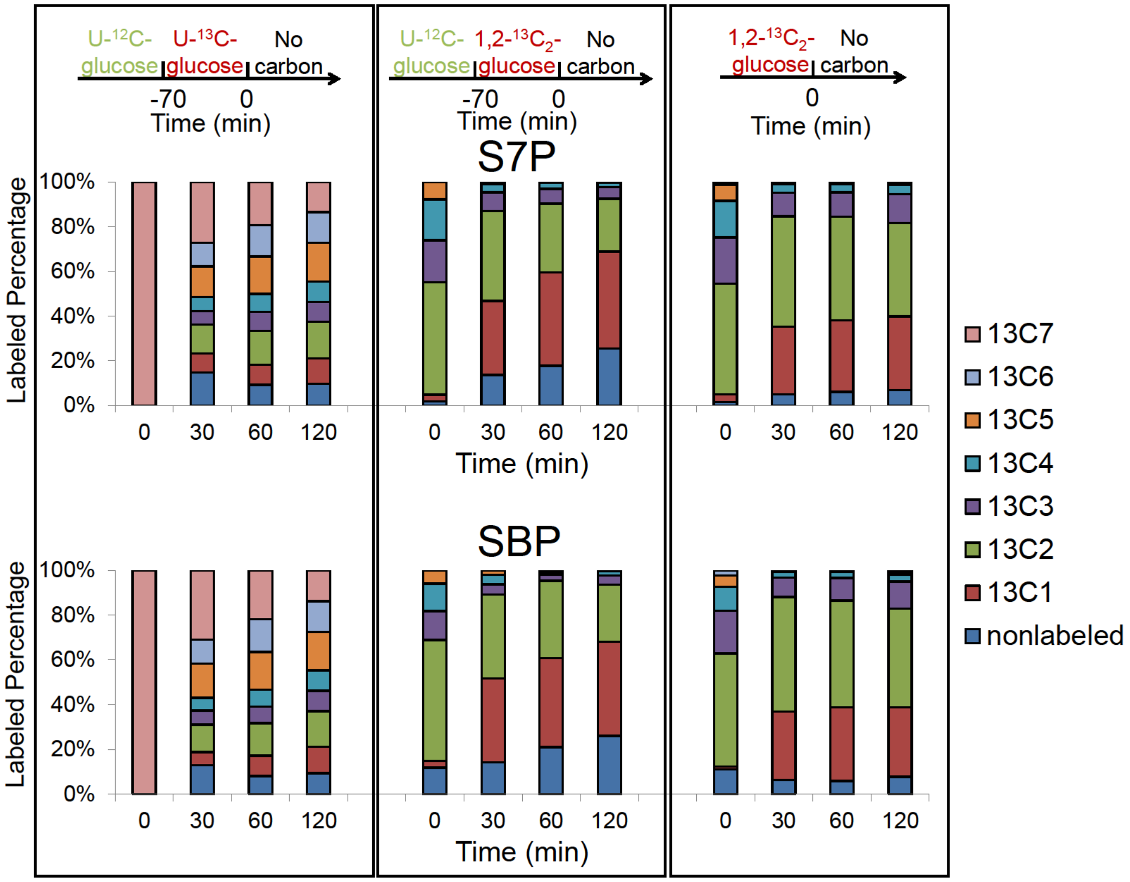

Figure S3. SBP was produced from S7P.

When feeding different labeled form of glucose before starvation, S7P and SBP exhibited identical labeling patterns in the following carbon starvation. The top panel of each block represents the experimental design of the data shown in the bottom panel of the same block. Left block: Yeast cells growing on unlabeled glucose were switched to U-^{13}C-glucose for 70 minutes. Thereafter, glucose was removed and metabolome analyzed by LC-MS. Middle block: Yeast cells growing on unlabeled glucose were switched to 1,2-$^{13}C_2$-glucose for 70 minutes. Thereafter, glucose was removed and metabolome analyzed by LC-MS. Right block: Yeast cells growing on 1,2-$^{13}C_2$-glucose for more than 10 generations were switched to no carbon and metabolome analyzed. The *y*-axis in the data represents percentage of each labeled form.

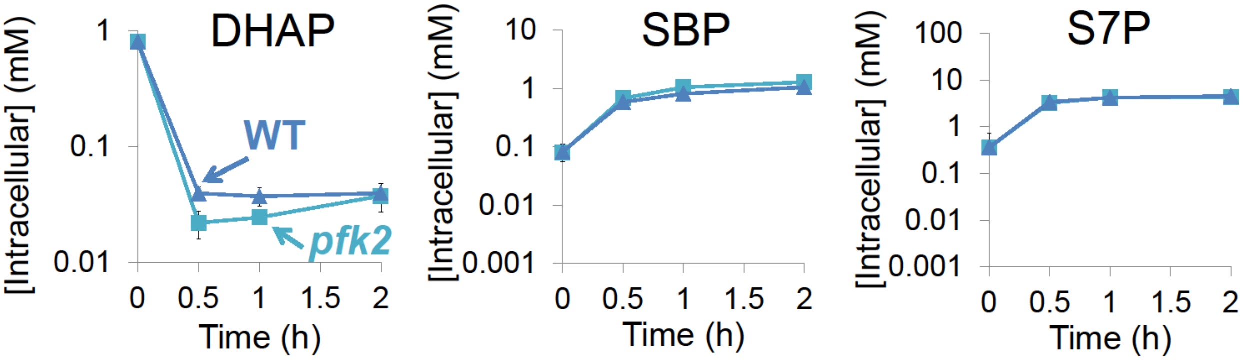

Figure S4. ***pfk2*** **deletion doesn't show changes of SBP and S7P**

DHAP, SBP and S7P levels in wild type and *pfk2* strains upon carbon starvation. The *x* axis represents hours after carbon starvation, and the logarithmic *y* axis represents absolute intracellular concentration (mean ± range of N = 2 biological replicates).

Table S1. Incorrect and missing annotations in the current genome scale metabolic model

Gene name	Systematic name	Description in the current genome scale model	Substrates in the current genome scale model	Annotations and corrections in this study
PHM8	YER037W	None		Major 5'-nucleotidase acting on GMP, UMP and CMP
SDT1	YGL224C	5'-nucleotidase	CMP, UMP	Minor 5'-nucleotidase (insufficient to substitute for *PHM8*)
PNP1	YLR209C	Nucleoside phosphorylase	adenosine, guanosine, inosine, xanthosine	No adenosine phosphorylase activity
URH1	YDR400W	Nucleoside hydrolase	adenosine, uridine, cytidine	No purine hydrolase activity
PGM3 (PRM15)	YMR278W	Phosphoglucomutase	glucose-1-phosphate, glucose-6-phosphate	Phosphoribomutase converting ribose-1-phosphate to ribose-5-phosphate
RBK1	YCR036W	Ribokinase	ribose	Minor ribokinase; other unknown major ribokinase exists

Table S2. Media used in nitrogen and phosphate starvation (Klosinska et al., 2011)

Comparison of salts composition in the media used

	**Minimal Media**	**Carbon starvation**	**Nitrogen limited**	**Nitrogen starvation**	**Phosphate limited**	**Phosphate starvation**
Glucose	20 g	-	20 g	20 g	20 g	20 g
Ammonium Sulfate	5 g	5 g	625 mg	-	5 g	5 g
Sodium Sulfate	-	-	5 g	5 g	-	-
Potassium phosphate monobasic	1 g	1 g	1 g	1 g	-	-
Potassium Chloride	-	-	-	-	1 g	1 g
Magnesium Sulfate	500 mg	500 mg	500 mg	500 mg	500 mg	500 mg
Sodium Chloride	100 mg	100 mg	100 mg	100 mg	100 mg	100 mg
Calcium Chloride	100 mg	100 mg	100 mg	100 mg	100 mg	100 mg
Vitamins and other metals	In yeast nitrogen base	In yeast nitrogen base	In yeast nitrogen base	In yeast nitrogen base	From 1000X (see below)	From 1000X (see below)

1000X vitamins and metals

**1000X vitamins (per 1L)**		**1000X Metals (per 1L)**	
Biotin	1 mg	Boric acid	500 mg
Calcium pantothenate	400 mg	Copper Sulfate.5H_2O	40 mg
Folic acid	2 mg	Potassium Iodide	100 mg
Myo-inositol	2000 mg	Ferric Chloride.6H_2O	200 mg
Nicotinic acid	400 mg	Manganese Sulfate.H_2O	400 mg
p-aminobenzoic acid	200 mg	Sodium Molybdate.2 H_2O	200 mg
Pyridoxine HCl	400 mg	Zinc Sulfate.7H_2O	400 mg
Riboflavin	200 mg		
Thiamine HCl	400 mg		

Table S3. Absolute concentration of metabolites in this study. Absolute concentrations were measured by an isotope ratio based method (Bennett et al., 2009) in a single condition where metabolites abundance is high. These direct measurements were shown in bold. Other values were inferred by fold change relative to the directly measured condition.

	Absolute concentration (mM) in replete condition (t = 0 for C-starvation)		Absolute concentration (mM) in carbon starvation (t = 1 h for C-starvation)	
Compound	Concentration	Standard Deviation	Concentration	Standard Deviation
AMP	**8.4E-02**	**1.7E-02**	1.2E-01	3.6E-02
CMP	**5.2E-03**	**2.0E-03**	1.8E-02	7.7E-05
DHAP	**8.1E-01**	**9.4E-02**	3.9E-02	9.2E-03
FBP	**4.0E+00**	**5.2E-01**	3.6E-02	3.1E-03
G6P	**5.9E+00**	**8.7E-01**	3.3E-01	1.8E-02
GMP	**1.0E-02**	**3.5E-03**	3.4E-02	2.0E-03
IMP	**3.8E-02**	**1.3E-02**	1.2E-02	1.4E-03
R5P	**1.5E-01**	**3.3E-02**	3.8E-02	8.7E-03
SBP	**8.2E-02**	**2.7E-02**	9.5E-01	1.9E-01
S7P	**3.6E-01**	**2.8E-02**	3.5E+00	8.1E-01
UMP	**1.5E-02**	**5.2E-03**	9.9E-02	1.5E-03
R1P	**1.5E-02**	**1.0E-03**	8.3E-03	3.7E-04
adenosine	3.3E-04	3.7E-05	**5.2E-03**	**3.4E-04**
cytidine	3.4E-04	9.6E-05	**9.0E-03**	**1.3E-03**
guanine	2.6E-03	0.0E+00	**1.5E-01**	**9.7E-03**
guanosine	3.3E-04	6.9E-05	**6.8E-03**	**3.7E-04**
hypoxanthine	4.4E-03	3.3E-04	**1.2E+00**	**9.0E-02**
inosine	3.2E-03	7.4E-05	**8.9E-03**	**7.1E-04**
uracil	3.7E-02	1.1E-02	**4.1E+00**	**7.2E-02**
uridine	7.2E-03	5.9E-04	**2.8E-01**	**1.3E-02**
xanthine	3.0E-02	9.1E-03	**1.8E+00**	**1.4E-01**

Supplemental Reference

Bennett, B.D., Kimball, E.H., Gao, M., Osterhout, R., Van Dien, S.J., and Rabinowitz, J.D. (2009). Absolute metabolite concentrations and implied enzyme active site occupancy in Escherichia coli. Nature Chemical Biology *5*, 593-599.

Klosinska, M.M., Crutchfield, C.A., Bradley, P.H., Rabinowitz, J.D., and Broach, J.R. (2011). Yeast cells can access distinct quiescent states. Gene Dev *25*, 336-349.
